# Association between hyperuricemia and the risk of mortality in patients with osteoarthritis: A study based on the National Health and Nutrition Examination Survey database

**DOI:** 10.1371/journal.pone.0302386

**Published:** 2024-05-07

**Authors:** Ye Hao, Xin Tang, Feng Xu

**Affiliations:** Articular Surgery, Beijing Shijingshan Hospital, Beijing, P.R. China; Federal Medical Centre Umuahia, NIGERIA

## Abstract

**Background:**

The purpose of this study was to evaluate the relationship between hyperuricemia and the risks of all-cause mortality and cardiovascular disease (CVD) mortality in patients with osteoarthritis (OA).

**Methods:**

A retrospective cohort study was performed on 3,971 patients using data from the National Health and Nutrition Examination Survey database between 1999 and 2018. OA was diagnosed through specific questions and responses. The weighted COX regression models were used to explore the factors associated with all-cause mortality/CVD mortality in OA patients. Subgroup analyses were conducted based on age, gender, hypertension, dyslipidemia, CVD, and chronic kidney disease (CKD). Hazard ratio (HR) and 95% confidence interval (95% CI) were measured as the evaluation indexes.

**Results:**

During the duration of follow-up time (116.38 ± 2.19 months), 33.69% (1,338 patients) experienced all-cause mortality, and 11.36% (451 patients) died from CVD. Hyperuricemia was associated with higher risks of all-cause mortality (HR: 1.22, 95% CI: 1.06–1.41, P = 0.008) and CVD mortality (HR: 1.32, 95% CI: 1.02–1.72, P = 0.036) in OA patients. Subgroup analyses showed that hyperuricemia was related to the risk of all-cause mortality in OA patients aged >65 years (HR: 1.17, 95% CI: 1.01–1.36, P = 0.042), in all male patients (HR: 1.41, 95% CI: 1.10–1.80, P = 0.006), those diagnosed with hypertension (HR: 1.17, 95% CI: 1.01–1.37, P = 0.049), dyslipidemia (HR: 1.18, 95% CI: 1.01–1.39, P = 0.041), CVD (HR: 1.30, 95% CI: 1.09–1.55, P = 0.004), and CKD (HR: 1.31, 95% CI: 1.01–1.70, P = 0.046). The association between hyperuricemia and a higher risk of CVD mortality was found in OA patients aged ≤ 65 years (HR: 1.90, 95% CI: 1.06–3.41, P = 0.032), who did not suffer from diabetes (HR: 1.36, 95% CI: 1.01–1.86, P = 0.048), who did not suffer from hypertension (HR: 2.56, 95% CI: 1.12–5.86, P = 0.026), and who did not suffer from dyslipidemia (HR: 2.39, 95% CI: 1.15–4.97, P = 0.020).

**Conclusion:**

These findings emphasize the importance of monitoring serum uric acid levels in OA patients for potentially reducing mortality associated with the disease.

## Introduction

Osteoarthritis (OA) is a chronic and degenerative joint disease and represents the most prevalent type of arthritis among adults [1, 2]. OA most commonly affects the joints of the hip, knee, and hand, but most joints can be affected [3]. As of 2020, approximately 595 million individuals globally were afflicted with OA, constituting about 7.6% of the world’s population [1]. OA is a primary cause of chronic pain and long-term disability in adults [4]. Furthermore, patients with OA exhibit a significantly increased risk of all-cause mortality as well as heightened susceptibility to cardiovascular disease (CVD) mortality [5, 6]. Therefore, identifying factors that influence the mortality risk in patients with OA is crucial for the prognostic management of these patients.

Hyperuricemia is a metabolic disorder caused by abnormal uric acid metabolism [7]. Elevated levels of uric acid in the blood can cause uric acid crystals to deposit in joints and other tissues, triggering an inflammatory response [8]. Inflammation also plays a crucial role in the progression of OA [9, 10]. Previous research has indicated that colchicine, a medication commonly used to treat hyperuricemia, can improve the inflammatory state in patients with OA [11]. Multiple studies have indicated a significant association between hyperuricemia and OA. In a study including 195 with knee OA, a significant association between asymptomatic hyperuricemia and knee OA in these patients was observed, even after adjusting for factors [12]. Hyperuricemia has also been found to be associated with a higher prevalence of hand OA [13]. In a study specifically examining knee OA, patients with higher serum uric acid levels displayed more severe symptoms and radiological signs of knee OA, including more severe pain, stiffness, and function scores [14]. However, there is a lack of research exploring the relationship between hyperuricemia and the overall mortality risk or the risk of CVD death in patients with OA from the literature search by the authors.

Herein, this study aims to investigate the association between hyperuricemia and the risks of all-cause mortality and CVD mortality in patients with OA. This study may provide scientific evidence to support prognosis management in OA patients.

## Methods

### Study design and participants selection

This study was a retrospective cohort study utilizing data from the National Health and Nutrition Examination Survey (NHANES) database from 1999 to 2018. The NHANES [15], conducted by the National Center of Health Statistics (NCHS) at the Centers for Disease Control and Prevention (CDC), is a comprehensive program aimed at evaluating the health and nutritional status of both adults and children in the United States. This survey provides extensive data on American health and nutrition, utilizing a multi-phase, probability sampling design, and detailed coding variables. The collected data encompass a wide range of health and nutrition-related topics, making it a valuable resource for health research and policy development. Patient records were extracted from the NHANES database for this study if they met the following criteria: 1) patients aged ≥ 18 years; 2) OA diagnosed by self-report refers to participants who have indicated a medical diagnosis of OA provided by healthcare professionals [16]; 3) patients who were with the measurements of uric acid. The exclusion criteria were as follows: 1) patients who were missing survival data; 2) patients who were missing important co-variables. The program of NHANES was reviewed and approved by the Prevention NCHS and the CDC Research Ethics Review Board, and all participants signed written informed consent.

### Definitions of OA and hyperuricemia

The NHANES identifies patients with OA through specific questions and responses. Patients were first asked if a doctor had ever told them they had arthritis (MCQ160a). If the answer was yes, they were further queried about the type of arthritis they have, with OA being one of the options (MCQ195/MCQ 190/MCQ191: Which type of arthritis was it?). Hyperuricemia was defined as ≥7 mg/dL in men and ≥6 mg/dL in women [17].

### Outcome and follow-up

The outcome focused on evaluating the association between hyperuricemia and two key outcomes: all-cause mortality and CVD mortality in patients with OA. The all-cause mortality refers to the death of an individual owing to various causes. CVD mortality was defined as deaths caused by CVD. Follow-up consisted of a physical examination or telephone interview. The follow-up process included physical examinations or telephone interviews, continuing until an outcome event occurred or until December 2019. The average duration of follow-up was approximately 116.38 ± 2.19 months.

### Potential covariates and definitions

In the study, age was categorized into two groups for analysis: age ≤ 65 years and age > 65 years. Gender distribution was categorized as male and female. Race included non-Hispanic White, non-Hispanic Black, and others. The educational levels of participants were classified into three categories: below high school, high school, college and above. The poverty-income ratio (PIR) was categorized into three groups: < 1.0, > = 1.0, and unknown. According to the questionnaire, those who smoked at least 100 cigarettes during their lifetime were regarded as smoking. The participants’ alcohol consumption is categorized based on frequency: less than 2 times per week and 2 or more times per week. Physical activity was converted into energy consumption based on the questionnaire in the database. Energy consumption [metabolic equivalents (MET) × min] = recommended MET × exercise time of corresponding activity (min), which can be converted into weekly energy consumption. Physical activity was divided into three categories < 450 MET·min/week, ≥450 MET·min/week, and Unknown. The mean total energy was assessed. The duration of arthritis was calculated as follows: age at screening minus age at which the patient received a diagnosis of arthritis.

Chronic kidney disease (CKD) was defined as the preoperative estimated glomerular filtration rate (eGFR; ml/min/1.73m^2^) < 60 [18]. Osteoporosis was defined with an affirmative response to the questions, “Has a doctor ever told you that you had osteoporosis, sometimes called thin or brittle bones?” or according to bone mineral density (BMD) T-score < = -2.5 at either lumbar spine or femoral neck. Fractures were determined based on participant’s responses to specific questions: OSQ010a-Broken or fractured a hip), OSQ010b-Broken or fractured a wrist, and OSQ010c-Broken or fractured spine. Liver disease was based on previous medical history, clinical and laboratory tests. Chronic obstructive pulmonary disease (COPD) was identified either through a specific question (MCQ160o), where participants are asked if a doctor ever told them they have COPD, or through medication codes (122–125). In terms of cancer, participants were asked the survey question “Have you ever been told by a doctor or other health professional that you had had cancer or a malignancy of any kind?”. CVD was defined based on the answer of “Yes” to variable MCQ160D (Ever told you had angina or heart failure?), MCQ160E (Ever told you had a heart attack?), MCQ160C (Has a doctor or other health professional ever told you that you had coronary heart disease?), MCQ160F (Ever told you had a stroke?), MCQ160B (Ever told had congestive heart failure?), or those received CVD drugs based on 40-CARDIOVASCULAR AGENTS-41, 43, 44, 45, 46, 50, 51, 52, 53, 54, 56, 303, 340, 342, 430, 433, 483. Dyslipidemia was defined based on total cholesterol (TC) ≥ 200 mg/dL (5.2 mmol/L) or triglyceride (TG) ≥ 150 mg/dL (1.7 mmol/L) or low-density lipoprotein cholesterol (LDL-C) ≥ 130 mg/dL (3.4 mmol/L) or high-density lipoprotein cholesterol (HDL-C) ≤ 40 mg/dL (1.0 mmol/L) [19], previous physician diagnosed hypercholesterolemia (BPQ080) or receiving cholesterol-lowering treatment (BPQ090D) or lipid-lowering drugs (358-metabolic agents-19-antihyperlipemic agents). Hypertension was defined as systolic blood pressure (SBP) ≥ 140 mmHg and or diastolic blood pressure (DBP) ≥ 90 mmHg or previous physician-diagnosed hypertension (BPQ020) or(BPQ030) or taking blood pressure medications (BPQ040A or drug code 40-CARDIOVASCULAR AGENTS-42, 47, 48, 49, 482, 55). Diabetes was diagnosed based on glycated hemoglobin ≥ 6.5%, fasting glucose ≥ 126 mg/dL, 2 h oral glucose tolerance test blood glucose ≥ 200 mg/dL, previous physician-diagnosed diabetes [DIQ010 (Doctor told you have diabetes)], insulin use (DIQ050) or antidiabetic agents (DIQ070 or 358-metabolic Agents-99-antidiabetic agents) [20]. The use of anti-hyperuricemic agents was determined by participants reporting the use of medications coded as 358-METABOLIC AGENTS-289-antihyperuricemic agents. Gout was defined as participants who answered “Yes” to the question, “Doctor ever told you that you had gout?” or through the identification of medication use with the code 358-METABOLIC AGENTS-194-ANTIGOUT AGENTS. White blood cell count was calculated. OA drug (No/Unknown, Yes) was included in this study.

### Statistical analysis

The measurement data were described by Mean (standard deviation) [Mean (SD)], the independent sample t-test was used for comparison between two groups, and the analysis of variance (ANOVA) was used for comparison between multiple groups. The enumeration data were described by the number of cases and composition ratio N (%), the Chi-square test was used for comparison between groups, and the rank sum test was applied for ranked data. For variables with a high rate of missing data (PIR, and physical activity), the missing values are categorized under “Unknown”. Missing data was imputed by multiple imputations by chained equations with a random forest-based method. The sensitivity analysis was used to compare datasets before and after imputations.

On the basis of adjusting the usual confounding factors including age, gender, and race, the remaining variables were screened out by the weighted COX univariate regression model, and the variables with P value <0.05 from the univariate analysis were taken together as covariates. The univariate and multivariable weighted COX regression models were used to explore the factors associated with all-cause mortality/CVD mortality in OA patients. Subgroup analyses were conducted based on age (≤ 65 years and > 65 years), gender (male and female), diabetes (yes or no), hypertension (yes or no), dyslipidemia (yes or no), CVD (yes or no), CKD (yes or no). Hazard ratio (HR) and 95% confidence interval (95% CI) were measured as the evaluation indexes. All statistical tests were conducted using two-tailed tests, with a significance level of α = 0.05. Data cleaning and handling of missing values were performed using Python 3.9. Model statistical analysis was carried out using SAS 9.4 (SAS Institute, NC, USA). The imputation of missing data was conducted using the ’miceforest’ package in Python.

## Results

### Basic characteristics of included participants

**Fig 1:**
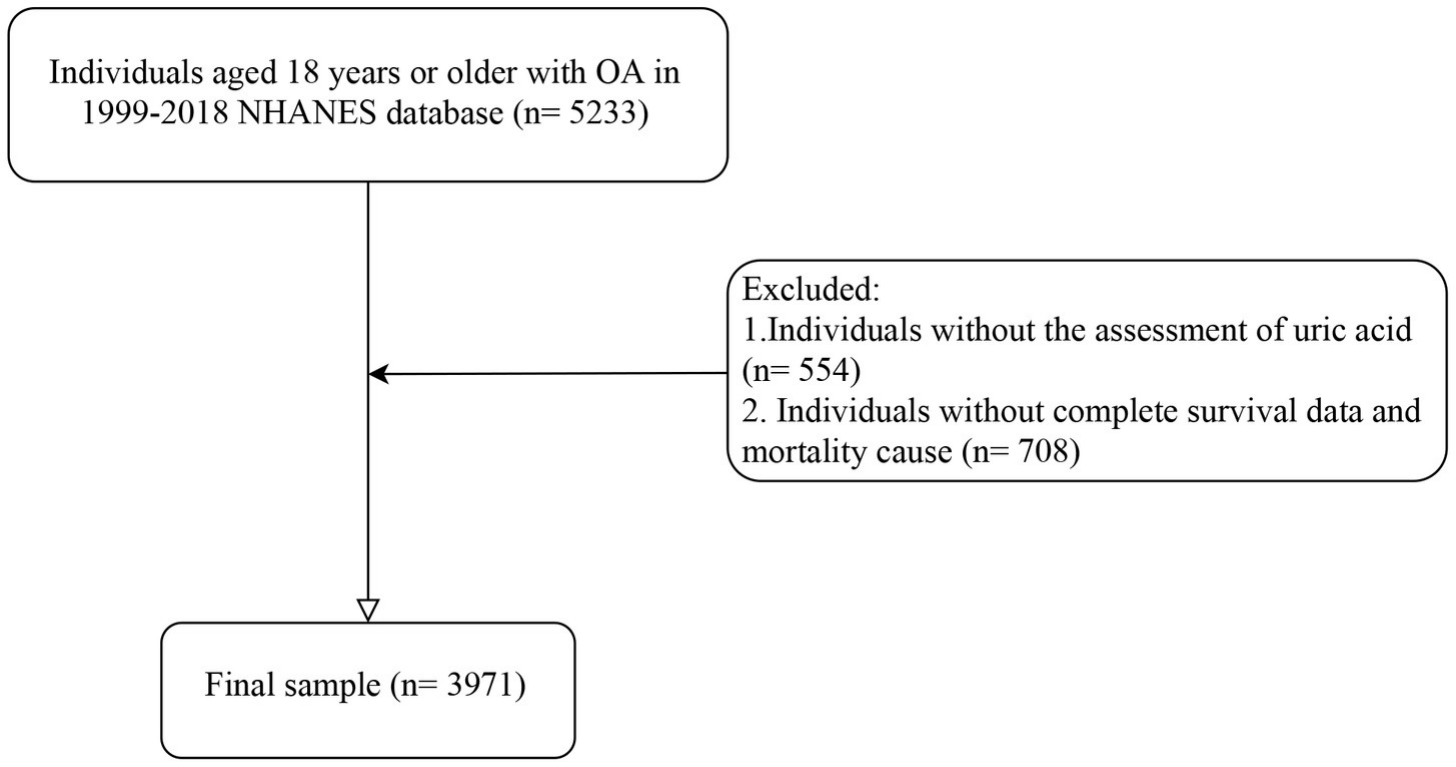
The flow chart of patients’ selection. OA, osteoarthritis; NHANES, National Health and Nutrition Examination Survey.

This study identified 5,233 individuals aged 18 years or older with OA from the NHANES database spanning 1999 to 2018. Following specific inclusion and exclusion criteria, 3,971 patients were selected for analysis. The study’s flowchart in Fig 1 details the patient selection process. Among these 3,971 patients, 33.69% (1,338 patients) experienced all-cause mortality, and 11.36% (451 patients) died from CVD. A comparison of the basic characteristics of alive and dead populations is presented in Table 1. Table 2 provides a comparison of the basic characteristics of patients with and without CVD deaths. The mean age of included patients was 61.94 (0.26) years. The mean uric acid level was 5.50 (0.03) mg/dL. There were 1,434 male participants (34.79%) and 2,537 female participants (65.21%). The racial composition of the study’s 3,971 participants was predominantly Non-Hispanic White (2,639 participants, 84.78%), followed by Others (783 participants, 9.37%), and Non-Hispanic Black (549 participants, 5.86%). In the study, 15.83% of participants (924 individuals) had an education level below high school, 22.49% (898 individuals) were high school graduates, and the majority, 61.68% (2,149 individuals), had a college education or higher. Totally, 82.42% of participants (3,173 individuals) did not have osteoporosis, while 17.58% (798 individuals) did. Additionally, 86.35% of participants (3,430 individuals) had no fractures, whereas 13.65% (541 individuals) had experienced fractures. A total of 1,053 participants (24.94%) had hyperuricemia. Of the included patients, 93.54% of participants (3,683 individuals) did not have gout, while 6.46% (288 individuals) were affected by gout. Significant differences and distributions between the alive and dead groups were observed in several aspects, including uric acid levels, hyperuricemia, age, race, education, PIR, smoking and alcohol status, physical activity, total energy intake, duration of arthritis, CKD, osteoporosis, fractures, COPD, cancer, family history of CVD, diabetes, hypertension, CVD, use of anti-hyperuricemic agents, white blood cell count, and follow-up time, all with P-values less than 0.05.

**Table 1:** Basic characteristics of alive and dead populations.

Variables	Total (n = 3971)	Alive (n = 2633)	Dead (n = 1338)	Statistics	*P*
Uric acid, mg/dL, Mean (S.E)	5.50 (0.03)	5.39 (0.04)	5.78 (0.05)	t = -6.28	<0.001
Hyperuricemia, n(%)				χ^2^ = 52.25	<0.001
No	2918 (75.06)	2017 (78.14)	901 (66.84)		
Yes	1053 (24.94)	616 (21.86)	437 (33.16)		
Age, years, Mean (S.E)	61.94 (0.26)	58.95 (0.30)	69.90 (0.39)	t = -22.16	<0.001
Age, years, n(%)				χ^2^ = 267.17	<0.001
≤ 65	1948 (57.12)	1619 (66.98)	329 0.87)		
> 65	2023 (42.88)	1014 (33.02)	1009 (69.13)		
Gender, n(%)				χ^2^ = 2.10	0.147
Male	1434 (34.79)	893 (34.01)	541 (36.88)		
Female	2537 (65.21)	1740 (65.99)	797 (63.12)		
Race, n(%)				χ^2^ = 8.04	0.018
Non-Hispanic White	2639 (84.78)	1643 (83.83)	996 (87.30)		
Non-Hispanic Black	549 (5.86)	393 (6.00)	156 (5.48)		
Others	783 (9.37)	597 (10.17)	186 (7.22)		
Education, n(%)				χ^2^ = 94.93	<0.001
Below high school	924 (15.83)	512 (12.18)	412 (25.56)		
High school	898 (22.49)	622 (22.95)	276 (21.26)		
College and above	2149 (61.68)	1499 (64.87)	650 (53.18)		
PIR, n(%)				χ^2^ = 8.58	0.014
< 1.0	573 (9.68)	398 (8.89)	175 (11.78)		
≥ 1.0	3116 (84.45)	2058 (85.57)	1058 (81.48)		
Unknown	282 (5.87)	177 (5.54)	105 (6.74)		
Smoking status, n(%)				χ^2^ = 6.78	0.009
No	1869 (47.11)	1288 (48.74)	581 (42.76)		
Yes	2102 (52.89)	1345 (51.26)	757 (57.24)		
Drinking status, n(%)				χ^2^ = 30.95	<0.001
< 2 times/week	1211 (27.91)	757 (25.16)	454 (35.48)		
≥ 2 times/week	2554 (72.09)	1756 (74.84)	798 (64.52)		
Physical activity, n(%)				χ^2^ = 209.87	<0.001
< 450 met*minutes/week	662 (17.52)	440 (17.81)	222 (16.72)		
≥ 450 met*minutes/week	1674 (46.84)	1315 (53.76)	359 (28.40)		
Unknown	1635 (35.65)	878 (28.43)	757 (54.88)		
Total energy, kcal, Mean (S.E)	1944.84 (17.19)	2008.28 (21.74)	1775.80 (24.19)	t = 7.24	<0.001
Duration of arthritis, years, Mean (S.E)	12.42 (0.23)	11.57 (0.27)	14.71 (0.47)	t = -5.58	<0.001
CKD, n(%)				χ^2^ = 166.57	<0.001
No	3535 (91.67)	2477 (95.34)	1058 (81.87)		
Yes	436 (8.33)	156 (4.66)	280 (18.13)		
Osteoporosis, n(%)				χ^2^ = 142.09	<0.001
No	3173 (82.42)	2238 (86.92)	935 (70.41)		
Yes	798 (17.58)	395 (13.08)	403 (29.59)		
Fracture, n(%)				χ^2^ = 29.38	<0.001
No	3430 (86.35)	2342 (88.52)	1088 (80.59)		
Yes	541 (13.65)	291 (11.48)	250 (19.41)		
Liver disease, n(%)				χ^2^ = 0.08	0.780
No	3732 (94.51)	2464 (94.58)	1268 (94.31)		
Yes	239 (5.49)	169 (5.42)	70 (5.69)		
COPD, n(%)				χ^2^ = 5.29	0.022
No	3395 (85.33)	2253 (86.29)	1142 (82.77)		
Yes	576 (14.67)	380 (13.71)	196 (17.23)		
Cancer, n(%)				χ^2^ = 16.35	<0.001
No	3192 (79.37)	2195 (81.31)	997 (74.20)		
Yes	779 (20.63)	438 (18.69)	341 (25.80)		
Family history of diabetes, n(%)				χ^2^ = 0.01	0.930
No	2122 (54.93)	1372 (54.98)	750 (54.79)		
Yes	1849 (45.07)	1261 (45.02)	588 (45.21)		
Family history of CVD, n(%)				χ^2^ = 9.81	0.002
No	3340 (83.10)	2177 (81.84)	1163 (86.47)		
Yes	631 (16.90)	456 (18.16)	175 (13.53)		
Diabetes, n(%)				χ^2^ = 23.41	<0.001
No	2958 (78.70)	2012 (80.92)	946 (72.78)		
Yes	1013 (21.30)	621 (19.08)	392 (27.22)		
Hypertension, n(%)				χ^2^ = 134.36	<0.001
No	1090 (32.25)	864 (37.48)	226 (18.34)		
Yes	2881 (67.75)	1769 (62.52)	1112 (81.66)		
Dyslipidemia, n(%)				χ^2^ = 1.00	0.317
No	590 (15.02)	395 (15.38)	195 (14.04)		
Yes	3381 (84.98)	2238 (84.62)	1143 (85.96)		
CVD, n(%)				χ^2^ = 104.51	<0.001
No	2356 (63.19)	1735 (68.56)	621 (48.87)		
Yes	1615 (36.81)	898 (31.44)	717 (51.13)		
Central obesity, n(%)				χ^2^ = 2.49	0.114
No	1091 (28.23)	679 (27.54)	412 (30.08)		
Yes	2880 (71.77)	1954 (72.46)	926 (69.92)		
Gout, n(%)				χ^2^ = 0.25	0.620
No	3683 (93.54)	2448 (93.67)	1235 (93.19)		
Yes	288 (6.46)	185 (6.33)	103 (6.81)		
Antihyperuricemic agents, n(%)				χ^2^ = 6.18	0.013
No	3862 (97.54)	2577 (98.01)	1285 (96.31)		
Yes	109 (2.46)	56 (1.99)	53 (3.69)		
White blood cell count, 1000 cells/uL, Mean (S.E)	7.28 (0.05)	7.21 (0.06)	7.46 (0.09)	t = -2.34	0.020
OA drug, n(%)				χ^2^ = 2.48	0.116
No/Unknown	3481 (88.08)	2273 (87.56)	1208 (89.47)		
Yes	490 (11.92)	360 (12.44)	130 (10.53)		
Follow-up time, months, Mean (S.E)	116.38 (2.19)	121.30 (2.70)	103.28 (2.59)	t = 5.53	<0.001

Notes: T, t-test; χ^2^, Chi-square test; S.E, standard error; PIR, poverty-income ratio; CKD, chronic kidney disease; COPD, chronic obstructive pulmonary disease; CVD, cardiovascular disease; OA, osteoarthritis.

**Table 2:** Basic characteristics of patients with and without CVD mortality.

Variables	Total (n = 3971)	CVD mortality		Statistics	*P*
		No (n = 3520)	Yes (n = 451)		
Uric acid, mg/dL, Mean (S.E)	5.50 (0.03)	5.46 (0.03)	5.97 (0.09)	t = -5.09	<0.001
Hyperuricemia, n(%)				χ^2^ = 25.49	<0.001
No	2918 (75.06)	2623 (76.19)	295 (63.50)		
Yes	1053 (24.94)	897 (23.81)	156 (36.50)		
Age, years, Mean (S.E)	61.94 (0.26)	61.09 (0.26)	70.65 (0.64)	t = -14.37	<0.001
Age, years, n(%)				χ^2^ = 90.75	<0.001
≤ 65	1948 (57.12)	1848 (59.74)	100 0.39)		
> 65	2023 (42.88)	1672 (40.26)	351 (69.61)		
Gender, n(%)				χ^2^ = 1.72	0.190
Male	1434 (34.79)	1244 (34.46)	190 (38.16)		
Female	2537 (65.21)	2276 (65.54)	261 (61.84)		
Race, n(%)				χ^2^ = 4.23	0.121
Non-Hispanic White	2639 (84.78)	2293 (84.50)	346 (87.55)		
Non-Hispanic Black	549 (5.86)	494 (5.85)	55 (5.91)		
Others	783 (9.37)	733 (9.64)	50 (6.54)		
Education, n(%)				χ^2^ = 42.29	<0.001
Below high school	924 (15.83)	780 (14.61)	144 (28.26)		
High school	898 (22.49)	802 (22.70)	96 (20.36)		
College and above	2149 (61.68)	1938 (62.69)	211 (51.38)		
PIR, n(%)				χ^2^ = 1.62	0.444
< 1.0	573 (9.68)	517 (9.52)	56 (11.27)		
≥ 1.0	3116 (84.45)	2750 (84.53)	366 (83.68)		
Unknown	282 (5.87)	253 (5.95)	29 (5.05)		
Smoke, n(%)				χ^2^ = 1.03	0.309
No	1869 (47.11)	1668 (47.38)	201 (44.34)		
Yes	2102 (52.89)	1852 (52.62)	250 (55.66)		
Drink, n(%)				χ^2^ = 7.74	0.005
< 2 times/week	3203 (77.34)	2827 (76.71)	376 (83.82)		
≥ 2 times/week	768 (22.66)	693 (23.29)	75 (16.18)		
Physical activity, n(%)				χ^2^ = 87.61	<0.001
< 450 met*minutes/week	662 (17.52)	585 (17.57)	77 (16.91)		
≥ 450 met*minutes/week	1674 (46.84)	1565 (48.92)	109 (25.54)		
Unknown	1635 (35.65)	1370 (33.51)	265 (57.55)		
Total energy, kcal, Mean (S.E)	1944.84 (17.19)	1966.58 (17.90)	1722.35 (46.90)	t = 5.01	<0.001
Duration of arthritis, years, Mean (S.E)	12.42 (0.23)	12.17 (0.23)	15.01 (0.88)	t = -3.17	0.002
CKD, n(%)				χ^2^ = 66.27	<0.001
No	3535 (91.67)	3191 (92.68)	344 (81.25)		
Yes	436 (8.33)	329 (7.32)	107 (18.75)		
Osteoporosis, n(%)				χ^2^ = 47.86	<0.001
No	3173 (82.42)	2866 (83.75)	307 (68.72)		
Yes	798 (17.58)	654 (16.25)	144 (31.28)		
Fracture, n(%)				χ^2^ = 21.60	<0.001
No	3430 (86.35)	3076 (87.23)	354 (77.35)		
Yes	541 (13.65)	444 (12.77)	97 (22.65)		
Liver disease, n(%)				χ^2^ = 2.87	0.090
No	3732 (94.51)	3297 (94.33)	435 (96.34)		
Yes	239 (5.49)	223 (5.67)	16 (3.66)		
COPD, n(%)				χ^2^ = 0.02	0.895
No	3395 (85.33)	2999 (85.30)	396 (85.61)		
Yes	576 (14.67)	521 (14.70)	55 (14.39)		
Cancer, n(%)				χ^2^ = 1.21	0.272
No	3192 (79.37)	2848 (79.64)	344 (76.64)		
Yes	779 (20.63)	672 (20.36)	107 (23.36)		
Family history of diabetes, n(%)				χ^2^ = 0.01	0.925
No	2122 (54.93)	1864 (54.90)	258 (55.19)		
Yes	1849 (45.07)	1656 (45.10)	193 (44.81)		
Family history of CVD, n(%)				χ^2^ = 3.56	0.059
No	3340 (83.10)	2946 (82.71)	394 (87.12)		
Yes	631 (16.90)	574 (17.29)	57 (12.88)		
Diabetes, n(%)				χ^2^ = 8.27	0.004
No	2958 (78.70)	2649 (79.35)	309 (72.04)		
Yes	1013 (21.30)	871 (20.65)	142 (27.96)		
Hypertension, n(%)				χ^2^ = 55.17	<0.001
No	1090 (32.25)	1036 (34.08)	54 (13.54)		
Yes	2881 (67.75)	2484 (65.92)	397 (86.46)		
Dyslipidemia, n(%)				χ^2^ = 3.36	0.067
No	590 (15.02)	536 (15.36)	54 (11.48)		
Yes	3381 (84.98)	2984 (84.64)	397 (88.52)		
CVD, n(%)				χ^2^ = 79.11	<0.001
No	2356 (63.19)	2198 (65.52)	158 (39.28)		
Yes	1615 (36.81)	1322 (34.48)	293 (60.72)		
Central obesity, n(%)				χ^2^ = 0.06	0.814
No	1091 (28.23)	965 (28.29)	126 (27.65)		
Yes	2880 (71.77)	2555 (71.71)	325 (72.35)		
Gout, n(%)				χ^2^ = 1.95	0.163
No	3683 (93.54)	3277 (93.70)	406 (91.93)		
Yes	288 (6.46)	243 (6.30)	45 (8.07)		
Antihyperuricemic agents, n(%)				χ^2^ = 4.24	0.039
No	3862 (97.54)	3434 (97.71)	428 (95.79)		
Yes	109 (2.46)	86 (2.29)	23 (4.21)		
White blood cell count, 1000 cells/uL, Mean (S.E)	7.28 (0.05)	7.26 (0.05)	7.43 (0.12)	t = -1.31	0.192
OA drug, n(%)				χ^2^ = 0.05	0.817
No/Unknown	3481 (88.08)	3075 (88.11)	406 (87.73)		
Yes	490 (11.92)	445 (11.89)	45 (12.27)		
Follow-up time, months, Mean (S.E)	116.38 (2.19)	117.56 (2.34)	104.29 (4.46)	t = 2.74	0.007

Notes: T, t-test; χ^2^, Chi-square test; S.E, standard error; PIR, poverty-income ratio; CKD, chronic kidney disease; COPD, chronic obstructive pulmonary disease; CVD, cardiovascular disease; OA, osteoarthritis.

### Associations between hyperuricemia and the risk of mortality in patients with OA

The result demonstrated that hyperuricemia was associated with higher risks of all-cause mortality (HR: 1.22, 95% CI: 1.06–1.41, P = 0.008) and CVD mortality (HR: 1.32, 95% CI: 1.02–1.72, P = 0.036) in OA patients. The associations between hyperuricemia and the risk of mortality in patients with OA are shown in Table 3.

**Table 3:** Associations between hyperuricemia and the risk of mortality in patients with OA.

Outcomes	Indicators	Model I		Model II		Model III	
		HR (95%CI)	*P*	HR (95%CI)	*P*	HR (95%CI)	*P*
All-cause mortality	Hyperuricemia						
	No	Ref.		Ref.		Ref.	
	Yes	1.62 (1.41–1.87)	<0.001	1.52 (1.32–1.75)	<0.001	1.22 (1.06–1.41)	0.008
CVD mortality	Hyperuricemia						
	No	Ref.		Ref.		Ref.	
	Yes	1.88 (1.47–2.39)	<0.001	1.75 (1.38–2.22)	<0.001	1.32 (1.02–1.72)	0.036

Notes: HR, hazard ratio; CI, confidence interval; CVD, cardiovascular disease; OA, osteoarthritis; Model I was an unadjusted model; Model II adjusted for age, gender, and race. Model III of all-cause mortality adjusted for age, gender, race, education, PIR, smoking status, physical activity, total energy, duration of arthritis, CKD, osteoporosis, fracture, COPD, cancer, CVD, diabetes, hypertension, dyslipidemia, white blood cell count, gout, and anti-hyperuricemic agents; Model III of CVD mortality adjusted for age, gender, race, education, drink, physical activity, total energy, duration of arthritis, CKD, osteoporosis, fracture, cancer, CVD, diabetes, hypertension, dyslipidemia, white blood cell count, gout, anti-hyperuricemic agents.

### Subgroup analyses of the associations between hyperuricemia and the risk of mortality in patients with OA

The subgroup analyses examining the associations between hyperuricemia and the risk of mortality in patients with OA are presented in Table 4. The results showed that hyperuricemia was related to the risk of all-cause mortality in OA patients aged >65 (HR: 1.17, 95% CI: 1.01–1.36, P = 0.042), who were male gender (HR: 1.41, 95% CI: 1.10–1.80, P = 0.006), those diagnosed with hypertension (HR: 1.17, 95% CI: 1.01–1.37, P = 0.049), dyslipidemia (HR: 1.18, 95% CI: 1.01–1.39, P = 0.041), CVD (HR: 1.30, 95% CI:1 .09–1.55, P = 0.004), and CKD (HR: 1.31, 95% CI: 1.01–1.70, P = 0.046). The association between hyperuricemia and a higher risk of CVD mortality was found in OA patients aged ≤ 65 years (HR: 1.90, 95% CI: 1.06–3.41, P = 0.032), who didn’t suffer from diabetes (HR: 1.36, 95% CI: 1.01–1.86, P = 0.048), who didn’t suffer from hypertension (HR: 2.56, 95% CI: 1.12–5.86, P = 0.026), and who didn’t suffer from dyslipidemia (HR: 2.39, 95% CI: 1.15–4.97, P = 0.020).

**Table 4:** Subgroup analyses of the associations between hyperuricemia and the risk of mortality in patients with OA.

Subgroups	All-cause mortality				CVD mortality			
	Model		Model		Model		Model	
	HR (95%CI)	*P*	OR (95%CI)	*P*				
Subgroup I: Age	Age ≤ 65 (n = 1948)		Age > 65 (n = 2023)		Age ≤ 65 (n = 1948)		Age > 65 (n = 2023)	
Hyperuricemia								
No	Ref		Ref		Ref		Ref	
Yes	1.23 (0.91–1.66)	0.169	1.17 (1.01–1.36)	0.042	1.90 (1.06–3.41)	0.032	1.10 (0.84–1.44)	0.494
Subgroup II: Gender	Male (n = 1434)		Female (n = 2537)		Male (n = 1434)		Female (n = 2537)	
Hyperuricemia								
No	Ref		Ref		Ref		Ref	
Yes	1.41 (1.10–1.80)	0.006	1.12 (0.94–1.33)	0.192	1.41 (0.90–2.22)	0.132	1.22 (0.88–1.70)	0.23
Subgroup III: Diabetes	No (n = 2958)		Yes (n = 1013)		No (n = 2958)		Yes (n = 1013)	
Hyperuricemia								
No	Ref		Ref		Ref		Ref	
Yes	1.16 (0.96–1.40)	0.115	1.27 (0.99–1.64)	0.058	1.36 (1.01–1.86)	0.048	1.20 (0.78–1.86)	0.399
Subgroup IV: Hypertension	No (n = 1090)		Yes (n = 2881)		No (n = 1090)		Yes (n = 2881)	
Hyperuricemia								
No	Ref		Ref		Ref		Ref	
Yes	1.41 (0.89–2.26)	0.145	1.17 (1.01–1.37)	0.049	2.56 (1.12–5.86)	0.026	1.21 (0.92–1.58)	0.166
Subgroup V: Dyslipidemia	No ((n = 590)		Yes (n = 3381)		No ((n = 590)		Yes (n = 3381)	
Hyperuricemia								
No	Ref		Ref		Ref		Ref	
Yes	1.41 (0.94–2.12)	0.097	1.18 (1.01–1.39)	0.041	2.39 (1.15–4.97)	0.02	1.23 (0.93–1.62)	0.139
Subgroup VI: CVD	No (n = 2356)		Yes (n = 1615)		No (n = 2356)		Yes (n = 1615)	
Hyperuricemia								
No	Ref		Ref		Ref		Ref	
Yes	1.15 (0.93–1.44)	0.197	1.30 (1.09–1.55)	0.004	1.49 (0.99–2.24)	0.057	1.23 (0.89–1.70)	0.202
Subgroup VII: CKD	No (n = 3535)		Yes (n = 436)		No (n = 3535)		Yes (n = 436)	
Hyperuricemia								
No	Ref		Ref		Ref		Ref	
Yes	1.17 (0.98–1.41)	0.09	1.31 (1.01–1.70)	0.046	1.27 (0.94–1.73)	0.121	1.37 (0.95–1.97)	0.087

Notes: CVD, cardiovascular disease; OA, osteoarthritis; CKD, chronic kidney disease; HR, hazard ratio; CI, confidence interval.

**All-cause mortality:** Subgroup I adjusted for gender, race, education, PIR, smoking status, physical activity, total energy, duration of arthritis, CKD, osteoporosis, fracture, COPD, cancer, CVD, diabetes, hypertension, dyslipidemia, white blood cell count, gout, and anti-hyperuricemic agents;

Subgroup II adjusted for age, race, education, PIR, smoking status, physical activity, total energy, duration of arthritis, CKD, osteoporosis, fracture, COPD, cancer, CVD, diabetes, hypertension, dyslipidemia, white blood cell count, gout, and anti-hyperuricemic agents;

Subgroup III adjusted for age, gender, race, education, PIR, smoking status, physical activity, total energy, duration of arthritis, CKD, osteoporosis, fracture, COPD, cancer, CVD, hypertension, dyslipidemia, white blood cell count, gout, and anti-hyperuricemic agents;

Subgroup IV adjusted for age, gender, race, education, PIR, smoking status, physical activity, total energy, duration of arthritis, CKD, osteoporosis, fracture, COPD, cancer, CVD, diabetes, dyslipidemia, white blood cell count, gout, and anti-hyperuricemic agents;

Subgroup V adjusted for age, gender, race, education, PIR, smoking status, physical activity, total energy, duration of arthritis, CKD, osteoporosis, fracture, COPD, cancer, CVD, diabetes, hypertension, white blood cell count, gout, and anti-hyperuricemic agents;

Subgroup VI adjusted for age, gender, race, education, PIR, smoking status, physical activity, total energy, duration of arthritis, CKD, osteoporosis, fracture, COPD, cancer, diabetes, hypertension, dyslipidemia, white blood cell count, gout, and anti-hyperuricemic agents;

Subgroup VII adjusted for age, gender, race, education, PIR, smoke, physical activity, total energy, duration of arthritis, osteoporosis, fracture, COPD, cancer, CVD, diabetes, hypertension, dyslipidemia, white blood cell count, gout, and anti-hyperuricemic agents;

**CVD mortality:** Subgroup I adjusted for gender, race, education, drinking status, physical activity, total energy, duration of arthritis, CKD, osteoporosis, fracture, cancer, CVD, diabetes, hypertension, dyslipidemia, white blood cell count, gout, and antihyperuricemic agents;

Subgroup II adjusted for age, race, education, drinking status, physical activity, total energy, duration of arthritis, CKD, osteoporosis, fracture, cancer, CVD, diabetes, hypertension, dyslipidemia, white blood cell count, gout, and anti-hyperuricemic agents;

Subgroup III adjusted for age, gender, race, education, drinking status, physical activity, total energy, duration of arthritis, CKD, osteoporosis, fracture, cancer, CVD, hypertension, dyslipidemia, white blood cell count, gout, and anti-hyperuricemic agents;

Subgroup IV adjusted for age, gender, race, education, drinking status, physical activity, total energy, duration of arthritis, CKD, osteoporosis, fracture, cancer, CVD, diabetes, dyslipidemia, white blood cell count, gout, and anti-hyperuricemic agents;

Subgroup V adjusted for age, gender, race, education, drinking status, physical activity, total energy, duration of arthritis, CKD, osteoporosis, fracture, cancer, CVD, diabetes, hypertension, white blood cell count, gout, and anti-hyperuricemic agents;

Subgroup VI adjusted for age, gender, race, education, drinking status, physical activity, total energy, duration of arthritis, CKD, osteoporosis, fracture, cancer, diabetes, hypertension, dyslipidemia, white blood cell count, gout, and anti-hyperuricemic agents;

Subgroup VII adjusted for age, gender, race, education, drinking status, physical activity, total energy, duration of arthritis, osteoporosis, fracture, cancer, CVD, diabetes, hypertension, dyslipidemia, white blood cell count, gout, and anti-hyperuricemic agents.

## Discussion

In a comprehensive analysis using a public database, we evaluated the relationship between hyperuricemia and the likelihood of death from any cause or specifically from CVD in patients suffering from OA. Our results revealed that in patients with OA, hyperuricemia was linked to an increased chance of dying from any cause and CVD. Moreover, the increased risk of death from any cause was particularly notable in OA patients over 65 years old, males, and those with conditions including hypertension, dyslipidemia, CVD, and CKD. Nevertheless, in OA patients, hyperuricemia was primarily associated with a greater risk of dying from CVD, particularly in those who were 65 or younger, and did not have diabetes, hypertension, or dyslipidemia.

High uric acid levels, or hyperuricemia, have been studied in relation to their impact on OA. Data from the Xiangya OA study reported that hyperuricemia was associated with a higher prevalence of hand OA [13]. In a survey of 3099 individuals aged 65 and older, hyperuricemia was associated with hand OA in women, and hyperuricemia was significantly related to knee OA in men [21]. One study focused on the association of serum uric acid with the clinical and radiological severity of knee OA and found that the group with higher serum uric acid exhibited more severe changes, including a higher frequency of Kellgren-Lawrence (KL) grade 4, grade 4 osteophytes, and narrower joint space width (JSW) [14]. Our findings revealed that in patients with OA, hyperuricemia was associated with an increased risk of all-cause mortality and CVD mortality. Various previous studies have reported the association of hyperuricemia/ higher uric acid levels with all-cause and CVD mortality in various patient populations including healthy individuals. A nationwide community-based cohort study found that both all-cause and CVD mortality were higher in men and women with high serum uric acid levels [22]. In a large cohort study of men and women, high uric acid levels were predictive of increased mortality [23]. A longitudinal Taiwanese cohort including 127,771 adults 65 years and older demonstrated that higher serum uric acid levels independently predict higher all-cause and CVD-related mortality in the elderly [24]. Higher uric acid concentrations were independently associated with death in individuals with CKD [25]. Hyperuricemia was also found to modestly increase the risk of coronary heart disease mortality [26]. Heart failure patients with hyperuricemia were more likely to experience all-cause mortality in the long-term follow-up [27]. Zhao et al.’s study concluded that there is a nonlinear relationship between serum uric acid concentrations and all-cause mortality in the American OA population [28]. More studies are warranted to explore whether hyperuricemia is a direct causal relationship between OA and mortality.

The mechanisms linking hyperuricemia to an increased risk of all-cause mortality in OA patients may be due to inflammation. High uric acid levels are known to activate the NLRP3 (Nacht, leucine-rich repeat, and pyrin domain-containing protein 3) inflammasome [29]. When activated, the NLRP3 inflammasome leads to the production of inflammatory cytokines such as IL-18 and IL-1β [30]. These cytokines play a significant role in the progression of OA and can exacerbate joint inflammation and damage [31]. Hyperuricemia can induce oxidative stress, which is implicated in the pathogenesis of CVD; oxidative stress can damage cellular components, including lipids, proteins, and DNA, leading to vascular injury and the progression of atherosclerosis [32]. Uric acid has also been shown to have direct effects on blood vessels, including the promotion of smooth muscle cell proliferation and the inhibition of endothelial progenitor cell function, both of which can contribute to the development and progression of atherosclerosis [33].

The subgroup analyses showed that hyperuricemia was related to the risk of all-cause mortality in OA patients aged >65 years. However, the association between hyperuricemia and a higher risk of CVD mortality was found in OA patients aged ≤ 65 years. A previous study reported that the age at hyperuricemia onset was identified as an important predictor of CVD and all-cause mortality risk, and the prediction was more powerful in those with a younger age of hyperuricemia onset [34]. Subgroup analyses provide valuable insights that could lead to more tailored approaches to the care of OA patients. The link between hyperuricemia and all-cause mortality in older OA patients suggests that maintaining optimal uric acid levels may be crucial for enhancing longevity in this demographic. This could lead to the recommendation of regular uric acid monitoring and appropriate interventions as part of geriatric care for OA patients. The finding that hyperuricemia is associated with an increased risk of CVD mortality in younger OA patients is particularly significant. It implies that even at a younger age, unmanaged hyperuricemia can be a substantial risk factor for CVD mortality, warranting early and aggressive management strategies to mitigate CVD mortality risks in this population. In this investigation, the link between hyperuricemia and all-cause mortality was observed exclusively in male patients with OA. The underlying reasons or mechanisms may involve gender-specific physiological or hormonal differences that influence the effects of uric acid on the body. Men have higher uric acid levels than women [35]. A study by Konta et al. found that a slight increase in serum uric acid levels was an independent risk factor for all-cause mortality in both men and women, and the threshold values of uric acid for mortality might be different for men and women [22]. We observed the association between hyperuricemia and all-cause mortality in OA patients with comorbidities of hypertension, dyslipidemia, CVD, and CKD. However, in OA patients, hyperuricemia was associated with a greater risk of dying from CVD, particularly in those who did not have diabetes, hypertension, or dyslipidemia. This finding suggests more studies to determine whether hyperuricemia is an independent risk factor or a mere bystander with the other risk factors for all-cause mortality in OA patients and highlights the complex interplay between comorbidities and CVD health in OA patients with hyperuricemia.

The correlation between hyperuricemia and increased risks of all-cause and CVD mortality in OA patients holds significant clinical implications. This association underscores the need for healthcare providers and patients to be aware of the potential negative impact of high uric acid levels on the prognosis of OA and cardiovascular health in OA patients. Understanding this relationship can assist clinicians in creating more comprehensive treatment plans that focus not only on joint health but also on cardiovascular well-being and overall pathological state. This approach may include monitoring and managing serum uric acid levels, lifestyle and dietary modifications, and the use of appropriate medications.

The strength of this study is its large sample size and long-term follow-up, ensuring sufficient cases of both all-cause and CVD mortality. The sample was obtained through a multi-stage complex sampling method, ensuring good representativeness. Additionally, the research accounted for various factors, including socioeconomic status, lifestyle elements, comorbidities, and other potential confounding factors, offering a comprehensive view of the impacts of hyperuricemia on all-cause and CVD mortality. Nevertheless, the present study had some limitations. Firstly, the study may include potential recall bias, as information on disease history and other factors was obtained through questionnaires. Secondly, due to database limitations, serum uric acid levels were measured only once, and the impact of their fluctuations on the mortality risk in OA patients requires further investigation. Third, it is important to acknowledge the potential impact of diagnostic criteria on the findings of our study. The use of self-reported OA diagnoses may not always accurately reflect the true prevalence of the condition, as this approach can be subject to individual interpretation and recall bias. Moreover, the diagnosis of OA by non-specialist physicians might include a broader spectrum of joint conditions, which could lead to an overestimation of the prevalence of OA in our study population. Given these considerations, our study underscores the need for further research using more precise diagnostic methods, such as radiographic assessments or specialist evaluations, to validate our findings and provide a more accurate representation of the relationship between hyperuricemia, OA, and associated mortality risks.

## Conclusion

In OA patients, hyperuricemia emerges as a significant risk factor for both all-cause and CVD mortality. These findings highlight the potential link between hyperuricemia and OA, suggesting that hyperuricemia could contribute to the mortality of OA. Therefore, individuals with OA should prioritize the prevention and management of hyperuricemia, including regular monitoring of their uric acid levels, to mitigate associated health risks.

## Supporting information

S1 Raw data The file of the "Raw-data" is the raw data set. (CSV)

S1 Dataset The file of the "impute-after" is the data set for the final analysis. (CSV)

## References

[1] Global, regional, and national burden of osteoarthritis, 1990–2020 and projections to 2050: a systematic analysis for the Global Burden of Disease Study 2021. Lancet Rheumatol. 2023;5(9):e508–e22. Epub 2023/09/07. doi: 10.1016/S2665-9913(23)00163-7 . 37675071 PMC10477960

[2] Hawker GA, King LK. The Burden of Osteoarthritis in Older Adults. Clin Geriatr Med. 2022;38(2):181–92. Epub 2022/04/13. doi: 10.1016/j.cger.2021.11.005 . 35410675

[3] Hunter DJ, Bierma-Zeinstra S. Osteoarthritis. Lancet. 2019;393(10182):1745–59. Epub 2019/04/30. doi: 10.1016/S0140-6736(19)30417-9 . 31034380

[4] Rice D, McNair P, Huysmans E, Letzen J, Finan P. Best Evidence Rehabilitation for Chronic Pain Part 5: Osteoarthritis. J Clin Med. 2019;8(11). Epub 2019/10/28. doi: 10.3390/jcm8111769 . 31652929 PMC6912819

[5] Constantino de Campos G, Mundi R, Whittington C, Toutounji MJ, Ngai W, Sheehan B. Osteoarthritis, mobility-related comorbidities and mortality: an overview of meta-analyses. Ther Adv Musculoskelet Dis. 2020;12:1759720x20981219. Epub 2021/01/26. doi: 10.1177/1759720X20981219 . 33488786 PMC7768583

[6] Oh M, Kim MY, So MW, Lim DH, Choi SJ, Lee JH, et al. Association between knee osteoarthritis and mortality: a serial propensity score-matched cohort study. Korean J Intern Med. 2023;38(6):923–33. Epub 2023/11/09. doi: 10.3904/kjim.2023.222 . 37939669 PMC10636544

[7] George C, Leslie SW, Minter DA. Hyperuricemia. StatPearls. Treasure Island (FL) ineligible companies. Disclosure: Stephen Leslie declares no relevant financial relationships with ineligible companies. Disclosure: David Minter declares no relevant financial relationships with ineligible companies.: StatPearls Publishing Copyright © 2023, StatPearls Publishing LLC.; 2023.

[8] Joosten LAB, Crişan TO, Bjornstad P, Johnson RJ. Asymptomatic hyperuricaemia: a silent activator of the innate immune system. Nat Rev Rheumatol. 2020;16(2):75–86. Epub 2019/12/12. doi: 10.1038/s41584-019-0334-3 . 31822862 PMC7075706

[9] Knights AJ, Redding SJ, Maerz T. Inflammation in osteoarthritis: the latest progress and ongoing challenges. Curr Opin Rheumatol. 2023;35(2):128–34. Epub 2023/01/26. doi: 10.1097/BOR.0000000000000923 . 36695054 PMC10821795

[10] Scanzello CR. Role of low-grade inflammation in osteoarthritis. Curr Opin Rheumatol. 2017;29(1):79–85. Epub 2016/10/19. doi: 10.1097/BOR.0000000000000353 . 27755180 PMC5565735

[11] Leung YY, Haaland B, Huebner JL, Wong SBS, Tjai M, Wang C, et al. Colchicine lack of effectiveness in symptom and inflammation modification in knee osteoarthritis (COLKOA): a randomized controlled trial. Osteoarthritis Cartilage. 2018;26(5):631–40. Epub 2018/02/10. doi: 10.1016/j.joca.2018.01.026 . 29426008

[12] Cao TN, Huynh KN, Tran HT, Nguyen MD. Association between asymptomatic hyperuricemia and knee osteoarthritis in older outpatients. Eur Rev Med Pharmacol Sci. 2022;26(18):6600–7. Epub 2022/10/06. doi: 10.26355/eurrev_202209_29760 . 36196710

[13] Zhu Y, Li J, Zhang Y, Zhang W, Doherty M, Yang Z, et al. Association between hyperuricaemia and hand osteoarthritis: data from the Xiangya Osteoarthritis Study. RMD Open. 2023;9(4). Epub 2023/12/06. doi: 10.1136/rmdopen-2023-003683 . 38053456 PMC10693871

[14] Bassiouni SARAK El Adalany MA, Abdelsalam M Gharbia OM. Association of serum uric acid with clinical and radiological severity of knee osteoarthritis in non-gouty patients. Egyptian Rheumatology and Rehabilitation. 2021;48(1):8. doi: 10.1186/s43166-020-00055-w

[15] Fain JA. NHANES. Diabetes Educ. 2017;43(2):151. Epub 2017/03/28. doi: 10.1177/0145721717698651 . 28340543

[16] Li Y, Zhu J, Fan J, Cai S, Fan C, Zhong Y, et al. Associations of urinary levels of phenols and parabens with osteoarthritis among US adults in NHANES 2005–2014. Ecotoxicol Environ Saf. 2020;192:110293. Epub 2020/02/12. doi: 10.1016/j.ecoenv.2020.110293 . 32045785

[17] Zhang YY, Qiu HB, Tian JW. Association Between Vitamin D and Hyperuricemia Among Adults in the United States. Front Nutr. 2020;7:592777. Epub 2020/12/18. doi: 10.3389/fnut.2020.592777 . 33330592 PMC7714933

[18] Forbes A, Gallagher H. Chronic kidney disease in adults: assessment and management. Clin Med (Lond). 2020;20(2):128–32. Epub 2020/03/14. doi: 10.7861/clinmed.cg.20.2 . 32165439 PMC7081794

[19] Jellinger PS, Smith DA, Mehta AE, Ganda O, Handelsman Y, Rodbard HW, et al. American Association of Clinical Endocrinologists’ Guidelines for Management of Dyslipidemia and Prevention of Atherosclerosis. Endocr Pract. 2012;18 Suppl 1:1–78. Epub 2012/04/24. doi: 10.4158/ep.18.s1.1 . 22522068

[20] McClure ST, Schlechter H, Oh S, White K, Wu B, Pilla SJ, et al. Dietary intake of adults with and without diabetes: results from NHANES 2013–2016. BMJ Open Diabetes Res Care. 2020;8(1). Epub 2020/10/26. doi: 10.1136/bmjdrc-2020-001681 . 33099509 PMC7590352

[21] Musacchio E, Perissinotto E, Sartori L, Veronese N, Punzi L, Zambon S, et al. Hyperuricemia, Cardiovascular Profile, and Comorbidity in Older Men and Women: The Pro.V.A. Study. Rejuvenation Res. 2017;20(1):42–9. Epub 2016/06/01. doi: 10.1089/rej.2016.1834 . 27241310

[22] Konta T, Ichikawa K, Kawasaki R, Fujimoto S, Iseki K, Moriyama T, et al. Association between serum uric acid levels and mortality: a nationwide community-based cohort study. Scientific Reports. 2020;10(1):6066. doi: 10.1038/s41598-020-63134-0 . 32269262 PMC7142123

[23] Cho SK, Chang Y, Kim I, Ryu S. U-Shaped Association Between Serum Uric Acid Level and Risk of Mortality: A Cohort Study. Arthritis Rheumatol. 2018;70(7):1122–32. Epub 2018/04/26. doi: 10.1002/art.40472 . 29694719

[24] Tseng WC, Chen YT, Ou SM, Shih CJ, Tarng DC. U-Shaped Association Between Serum Uric Acid Levels With Cardiovascular and All-Cause Mortality in the Elderly: The Role of Malnourishment. J Am Heart Assoc. 2018;7(4). Epub 2018/02/15. doi: 10.1161/JAHA.117.007523 . 29440009 PMC5850189

[25] Srivastava A, Kaze AD, McMullan CJ, Isakova T, Waikar SS. Uric Acid and the Risks of Kidney Failure and Death in Individuals With CKD. Am J Kidney Dis. 2018;71(3):362–70. Epub 2017/11/15. doi: 10.1053/j.ajkd.2017.08.017 . 29132945 PMC5828916

[26] Zuo T, Liu X, Jiang L, Mao S, Yin X, Guo L. Hyperuricemia and coronary heart disease mortality: a meta-analysis of prospective cohort studies. BMC Cardiovasc Disord. 2016;16(1):207. Epub 2016/10/30. doi: 10.1186/s12872-016-0379-z . 27793095 PMC5084405

[27] Han Y, Cao Y, Han X, Di H, Yin Y, Wu J, et al. Hyperuricemia and gout increased the risk of long-term mortality in patients with heart failure: insights from the National Health and Nutrition Examination Survey. J Transl Med. 2023;21(1):463. Epub 2023/07/13. doi: 10.1186/s12967-023-04307-z . 37438830 PMC10339518

[28] Zhao J, Sha B, Zeng L, Dou Y, Huang H, Liang G, et al. J-shaped association of serum uric acid concentrations with all-cause mortality in individuals with osteoarthritis: A prospective cohort study. Joint Bone Spine. 2023;91(3):105679. Online ahead of print. doi: 10.1016/j.jbspin.2023.105679 . 38143017

[29] Denoble AE, Huffman KM, Stabler TV, Kelly SJ, Hershfield MS, McDaniel GE, et al. Uric acid is a danger signal of increasing risk for osteoarthritis through inflammasome activation. Proc Natl Acad Sci U S A. 2011;108(5):2088–93. Epub 2011/01/20. doi: 10.1073/pnas.1012743108 . 21245324 PMC3033282

[30] Huang Y, Xu W, Zhou R. NLRP3 inflammasome activation and cell death. Cell Mol Immunol. 2021;18(9):2114–27. Epub 2021/07/30. doi: 10.1038/s41423-021-00740-6 . 34321623 PMC8429580

[31] McAllister MJ, Chemaly M, Eakin AJ, Gibson DS, McGilligan VE. NLRP3 as a potentially novel biomarker for the management of osteoarthritis. Osteoarthritis Cartilage. 2018;26(5):612–9. Epub 2018/03/03. doi: 10.1016/j.joca.2018.02.901 . 29499288

[32] Nishizawa H, Maeda N, Shimomura I. Impact of hyperuricemia on chronic kidney disease and atherosclerotic cardiovascular disease. Hypertens Res. 2022; 45(4):635–40. Epub 2022/1/19. doi: 10.1038/s41440-021-00840-w . 35046512

[33] Borghi C, Domienik-Karłowicz J, Tykarski A, Widecka K, Filipiak KJ, Jaguszewski MJ, et al. Expert consensus for the diagnosis and treatment of patient with hyperuricemia and high cardiovascular risk: 2021 update. Cardiol J. 2021;28(1):1–14. doi: 10.5603/CJ.a2021.0001 . 33438180 PMC8105060

[34] Li L, Zhao M, Wang C, Zhang S, Yun C, Chen S, et al. Early onset of hyperuricemia is associated with increased cardiovascular disease and mortality risk. Clin Res Cardiol. 2021;110(7):1096–105. Epub 2021/04/14. doi: 10.1007/s00392-021-01849-4 . 33846840

[35] Wang YF, Li JX, Sun XS, Lai R, Sheng WL. High serum uric acid levels are a protective factor against unfavourable neurological functional outcome in patients with ischaemic stroke. J Int Med Res. 2018;46(5):1826–38. Epub 2018/03/14. doi: 10.1177/0300060517752996 29529907 PMC5991245

